# Study on an Integral Algorithm of Load Identification Based on Displacement Response

**DOI:** 10.3390/s21196403

**Published:** 2021-09-25

**Authors:** Xun Xu, Yashan Zhu, Kejing Tian, Tingcan Lin, Yunyu Li

**Affiliations:** 1 School of Civil Engineering and Architecture, Wuhan University of Technology, Wuhan 430070, China; xuxun@whut.edu.cn (X.X.); yashan@whut.edu.cn (Y.Z.); kejingtian@whut.edu.cn (K.T.); lintingcan@whut.edu.cn (T.L.); 2 School of Transportation and Logistics Engineering, Wuhan University of Technology, Wuhan 430070, China

**Keywords:** load identification, integral algorithm, basis function type, noise robustness

## Abstract

Load identification is a very important and challenging indirect load measurement method because load identification is an inverse problem solution with ill-conditioned characteristics. A new method of load identification is proposed here, in which a virtual function was introduced to establish integral structure equations of motion, and partial integration was applied to reduce the response types in the equations. The effects of loading duration, the type of basis function, and the number of basis function expansion items on the calculation efficiency and the accuracy of load identification were comprehensively taken into account. Numerical simulation and experimental results showed that our algorithm could not only effectively identify periodic and random loads, but there was also a trade-off between the calculation efficiency and identification accuracy. Additionally, our algorithm can improve the ill-conditionedness of the solution of load identification equations, has better robustness to noise, and has high computational efficiency.

## 1. Introduction

Load identification, which plays an important role in structural dynamic optimization, vibration control, structural damage diagnosis, structural maintenance, and other aspects, is an important research aspect in structural health monitoring. Direct load measurement may not be easy or directly achievable because of factors such as an inconvenient or indirectly accessible layout of sensors; uncertain loading time or positions; and insufficient durability of sensors whose structures are under operation or harsh environments such as acidic, alkaline, or high-salt environments and high temperature or pressure. However, it is more convenient to measure the structural responses. Thus, researchers have proposed a structural response-based load identification method.

Reasonable parameterized modeling of any load can highlight its main characteristics and significantly reduce the unknown quantities of loads to be identified. Researchers have fitted loads by means of various basis functions according to the load types. Wang et al. considered the load consistency of the left and right feet while walking, regarded the walking load as a periodic process based on a single step, and expressed the vertical continuous walking load by Fourier series so that control parameters such as dynamic load factor, phase, and walking frequency of the walking load could be characterized [1]. Obata et al. regarded six parameters (contact time, heel period, heel impact coefficient, tip of toe period, tip of toe impact coefficient, and impact of heel to tip of toe) as identification parameters based on the human walking force model to establish an objective function by means of the ratio of calculated and measured accelerations. The identification was performed by means of the GA algorithm [2]. The periodic characteristics of the walking load were clear. Fourier series have been applied for modeling, and the number of model orders generally does not exceed five. Liu et al. expressed the shield-surrounding rock interaction loads of a tunnel boring machine by means of circumferential and axial node parameters and shape functions in cylindrical coordinates [3]. This type of load modeling mainly considers the symmetry of the load to reduce the model parameters. Impact loads are relatively special for structural load identification, and basic parameters of their models include loading duration, loading and unloading time, and maximum load. Yan et al. expressed impact loads by means of Gaussian kernel functions [4]. Tan et al. applied trigonometric functions to model impact loads [5]. These types of load modeling methods take load characteristics (such as periodicity, symmetry, and short duration) into account to improve their calculation efficiencies and achieve better recognition effects. As for general loads, such as random loads, the load characteristics are unknown or insignificant so that it is necessary to study a reasonable random load modeling method. For this reason, much research has been carried out on the modeling of random loads. He et al. represented the loads to be identified by means of the second-generation Lagrangian wavelet [6,7]. Lei et al. applied the Daubechies wavelet to fit spatially distributed dynamic loads [8]. Xu et al. fitted the nonlinear restoring force with the Chebyshev polynomial model [9,10]. Zhang et al. proposed the expression of the load history by means of the load shape function based on the finite element shape function idea [11]. The research results show that the above-mentioned random load modeling methods can effectively identify loads, but there is a lack of corresponding research on the trade-off of their calculation efficiencies and identification accuracies. Thus, this was a focus of our study; namely, loads were fitted by means of various basis functions, and the reasonable number of basis function expansion items was determined in the case of the load duration under consideration. 

It is very difficult to directly solve the inverse problem of differential equations. Transformation of structural motion equations is crucial for load identification. Structural responses are the convolution of external loads and their impulse response functions. Discretization of a convolution integral into linear equations is a common method. Liu et al. identified impact loads by means of an inverse impulse response function [12], and Wang et al. constructed the inverse impulse response function and took the structure acceleration responses as inputs to identify the wheel–rail force of a running vehicle [13]. The principle of the inverse impulse response function method is intuitive, which requires that the initial response value starts at time 0 and generally uses a higher frequency and a large equation dimension, so its calculation efficiency may be low. In a load identification algorithm, measurements and residual calculations are commonly used as its objective function. Loads are identified by means of direct solution or optimization. Liu et al. established an objective function with strain measurements of the shield and the calculated errors and directly solved the load parameters with the Moore–Penrose generalized inverse solution [3]. Yu et al. applied the Newmark-β method to derive the relationship between the structural displacement responses and the external load function, where residuals of the structural displacement response were the objective function, and the external loads were solved by means of the principle of least square [14]. The Moore–Penrose generalized inverse solution or least square method is applied when the matrix condition number is good or when the signal-to-noise ratio is high. Otherwise, the optimization method can be used to solve the problem. Wang et al. constructed the functional relationship between structural displacement power spectrum, displacement duration, and walking loads and identified characteristic parameters of walking loads by means of the genetic algorithm [1]. Yang et al. put forward a genetic algorithm based only on a single point response (displacement, velocity, or acceleration) to identify loads [15]. He et al. [7], Miao et al. [16], Yang et al. [17], and Qiao et al. [18] took two norms (structural response measurements and calculations) as the objective function to establish load identification methods. Tan et al. established an objective function of residuals of acceleration measurement and calculation responses by means of the least square principle and applied the optimal algorithm of bird propagation to identify external loads [5]. Liu et al. neglected the weighted integral value of residuals between the structural response measurements and calculations in the load identification interval to establish load identification equations [19]. Optimization can remove the need for the direct inversion of a matrix. If the degree of non-linearity in the solution of a problem is high, the optimization algorithm is required to have a strong global optimization ability. As for a linear system, uncoupled motion equations of its modal domain structure system can be obtained through modal transformation [20]. Chen et al. put forward an expansion of structural modal coordinates and responses under the same basis function and utilized the relationship between structural responses and modal coordinates to calculate out the modal coordinate coefficient matrix [21]; moreover, the speed and acceleration of the structure could be calculated, and they were then brought into the structural motion equations to obtain the external loads. For the identification of loads in a modal domain, the modal order greatly affects identification effects. Xu et al. first identified the shape of external loads based on load independence in view of independent external loads and then applied the homogeneity of the linear system to identify their amplitudes [22]. For correlated multi-source loads or only one load, such a method has certain limitations. Li et al. [23] and Maes et al. [24] applied the local vibration test method to identify axial forces of Euler–Bernoulli and Timoshenko beams, respectively, according to beam modal characteristics. The beam end constraints are not necessary for this method. Zhou et al. obtained the structural displacement and strain modes by means of experiments and a finite element numerical simulation and established the functional relationship between strain and displacement based on their same modal coordinates so that a method could be proposed to identify transient loads on the basis of strain responses [25]. Maes et al. [26] modified the structural finite element model by means of the measured footbridge data and regarded the unknown random force as noise. The bridge deck impact and harmonic loads were identified by means of the joint input-state estimation algorithm. Bao et al. used acceleration responses to identify the cable vibration frequency and the cable force on the basis of the functional relationship between the cable vibration frequency and the tensile force [27]. The identification of the cable vibration frequency and the functional relationship between the cable frequency and tension greatly affected the identification of cable forces. Wang et al. [28], Li et al. [29], and Yan et al. [30] established the conditional probability function of acceleration responses and load identification parameters in the state space and proposed a load identification method based on a Bayesian frame model. Kalman filtering is a typical effective state estimation method. Li et al. [17], Zhang et al. [31], Yi et al. [32], Fan et al. [33], and Zhi et al. [34] regarded load identification parameters as an extended state vector to propose load identification algorithms based on extended Kalman filtering. While many parameters are identified by means of the Kalman filter method, it is difficult for the algorithm to converge to the true values; moreover, those short-duration loads are not easily identified.

Factors such as the ill-condition of load identification equations, measurement noise, and model error can affect the load identification accuracy. Normalization methods [13,16,35] can effectively improve the stability of the equation solution, but some useful information of loads is lost. Because of the difference in the matrix condition numbers during a load identification process, the optimization algorithms [5,35] can be applied to avoid inverting the matrix. Noise problems can be denoised by filtering methods. Sun et al. utilized the sensitivity of the measured strain identification load to temperature and proposed a time-varying average method to correct strain measurements [36]. For filtering the high-frequency components of measurement noise, Gao et al. proposed a multiple integral moving average method for the measurement responses by selecting an appropriate fixed weight function [37]. In view of the great effects of the model accuracy on load identification for complex structural components, Xue et al. proposed the modeling of centrifugal compressor impellers by means of the Hermitian wavelet shell elements to improve the accuracy of load identification [38]. On the basis of the sensitivity of the numerical iteration load identification algorithm to the initial value of the identification force, Xu et al. analyzed the reasons for the inaccuracy of the initial system values by means of the quasi-static method and proposed the application of the dichotomy and golden section method to iteratively modify the initial load values solved by means of the quasi-static method [39]. Because of the sensitivity of an inverse problem to noise, initial values, and model errors, identical transformation should be maintained for the derivation of an algorithm, and the algorithm should have a certain degree of robustness to noise.

There are many types of loads in actual projects, and their characteristics differ. The type of basis function and the number of expansion items for the parametric modeling of loads affect the effects of load identification. They should be further explored and resolved for load identification. The load identification equations can be derived from the structural motion differential equations. Because of the test incompleteness, excessive response types will increase the number of unknown parameters and the complexity of the solution. The response types can be reduced by means of the differential or difference method to introduce calculation errors and cause sensitivity to noise. An integral equation method for load identification is proposed here to solve the above issues.

## 2. The Principles of Our Load Identification Integration Algorithm

### 2.1. Structural Motion Integral Equation in a Weakly Equivalent Form

The motion equation of a structure under external loads is expressed as

(1) $$M\ddot{X}\left(t\right)+C\dot{X}\left(t\right)+KX\left(t\right)=F\left(t\right)$$

where M,C and K represent the N×N-dimension structural mass, damping, and stiffness matrices, respectively; $\ddot{X}\left(t\right),\dot{X}\left(t\right)$, and $X\left(t\right)$ represent the acceleration, velocity, and displacement vectors, respectively, whose forms are as follows:

(2a) $$\ddot{X}\left(t\right)={\left\{{\ddot{x}}_{1}\left(t\right),{\ddot{x}}_{2}\left(t\right),\cdots ,{\ddot{x}}_{i}\left(t\right),\cdots ,{\ddot{x}}_{N}\left(t\right)\right\}}^{T}$$

(2b) $$\dot{X}\left(t\right)={\left\{{\dot{x}}_{1}\left(t\right),{\dot{x}}_{2}\left(t\right),\cdots ,{\dot{x}}_{i}\left(t\right),\cdots ,{\dot{x}}_{N}\left(t\right)\right\}}^{T}$$

(2c) $$X\left(t\right)={\left\{x_{1}\left(t\right),x_{2}\left(t\right),\cdots ,x_{i}\left(t\right),\cdots ,x_{N}\left(t\right)\right\}}^{T}$$

$F\left(t\right)$ represents an N×1-dimension external load vector, which is expressed as

(2d) $$F\left(t\right)={\left\{f_{1}\left(t\right),f_{2}\left(t\right),\cdots ,f_{i}\left(t\right),\cdots ,f_{N}\left(t\right)\right\}}^{T}$$

For any virtual function matrix ($D\left(t\right)$), the following equation is always valid in the time sub-interval ($\left[t_{b}∼t_{e}\right]$), and Equations (1) and (2) are equivalent.

(3) $$\int_{t_{b}}^{t_{e}}D\left(t\right)\left[M\ddot{X}\left(t\right)+C\dot{X}\left(t\right)+KX\left(t\right)\right]dt=\int_{t_{b}}^{t_{e}}D\left(t\right)F\left(t\right)dt$$

**Figure 1:**
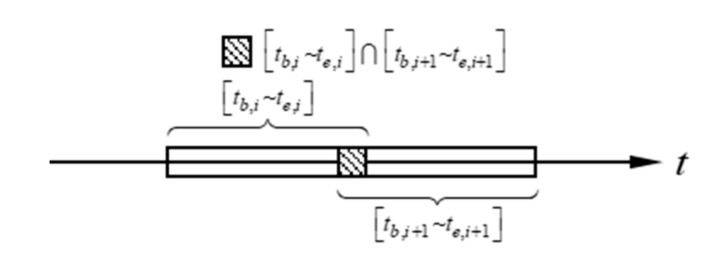
Division of time domain.

A load duration ($\left[0∼t\right]$) can be divided into $n_{t}$ time sub-intervals ($\left[t_{b,i}∼t_{e,i}\right]\left(i=1,2,\cdots ,n_{t}\right)$), all of which can fully cover $\left[0∼t\right]$. Adjacent time sub-intervals can be overlapped. As shown in Figure 1, their overlapping coefficient $\tau =\left[t_{b,i}\sim t_{e,i}\right]\cap \left[t_{b,i+1}\sim t_{e,i+1}\right]$. $\left[t_{b,i}∼t_{e,i}\right]$ is shorter, which reduces the number of calculations in this interval.

If $D\left(t\right)$ satisfies at least the second-order derivability, the partial integration of Equation (3) results in

(4) $${\left.\left[D\left(t\right)M\dot{X}\left(t\right)-\dot{D}\left(t\right)MX\left(t\right)+D\left(t\right)CX\left(t\right)\right]\right|}_{t_{b}}^{t_{e}}+\int_{t_{b}}^{t_{e}}\left[\ddot{D}\left(t\right)M-\dot{D}\left(t\right)C+D\left(t\right)K\right]X\left(t\right)dt=\int_{t_{b}}^{t_{e}}D\left(t\right)F\left(t\right)dt$$

In the actual solution process, the number $D\left(t\right)$ can only be limited for selection. Thus, Equations (1) and (4) are weakly equivalent. Equation (4) only contains $\dot{X}\left(t\right)$ and $X\left(t\right)$ of a structure.

Element (*i*, *j*) of $D\left(t\right)$ is expressed as $d_{ij}\left(t\right)$ ($i=1,2,\cdots ,N_{D}\left(N_{D}\geq N\right)$, j=1,2,⋯,N). In the case of $N_{D}=N$, $D\left(t\right)$ is a square matrix. Assuming $d_{ij}\left(t\right)\neq 0$, then

(5) $$D\left(t\right)=\left[\begin{array}{cccc} d_{11}\left(t\right) & d_{12}\left(t\right) & \cdots & d_{1N}\left(t\right)\\ d_{21}\left(t\right) & d_{22}\left(t\right) & \cdots & d_{2N}\left(t\right)\\ \vdots & \vdots & \ddots & \vdots \\ d_{N1}\left(t\right) & d_{N2}\left(t\right) & \cdots & d_{\mathrm{NN}}\left(t\right) \end{array}\right]$$

### 2.2. Parametric Expression of Structural Responses and External Loads

The parametric expression of structural responses and external loads can reduce the number of unknowns to be solved. $x_{i}\left(t\right)$ and $f_{i}\left(t\right)$ of the *i*^th^ degree of freedom are expanded under the basis function as

(6) $$x_{i}\left(t\right)=\xi_{i}\left(t\right)\alpha_{i}$$

where $\xi_{i}\left(t\right)=\left\{\xi_{i1}\left(t\right),\xi_{i2}\left(t\right),\cdots ,\xi_{ip}\left(t\right)\right\}$ represents the expanded basis function of $x_{i}\left(t\right)$, p represents the number of expansion terms of the basis function, and $\alpha_{i}\left(t\right)={\left\{\alpha_{i1},\alpha_{i2},\cdots ,\alpha_{ip}\right\}}^{T}$ represents the expanded combination coefficient of the displacement response.

(7) $$f_{i}\left(t\right)=\zeta_{i}\left(t\right)\beta_{i}$$

where $\zeta_{i}\left(t\right)=\left\{\zeta_{i1}\left(t\right),\zeta_{i2}\left(t\right),\cdots ,\zeta_{iq}\left(t\right)\right\}$ represents the expanded basis function of $f_{i}\left(t\right)$, q represents the number of expansion terms of the basis function, and $\beta_{i}\left(t\right)={\left\{\beta_{i1},\beta_{i2},\cdots ,\beta_{iq}\right\}}^{T}$ represents the expanded combination coefficient of the external load.

The same basis function and number of expansion items are assumed for the displacement responses and external loads, namely,

(8) $$\left\{\begin{array}{l} \xi_{1}\left(t\right)=\xi_{2}\left(t\right)=\cdots =\xi_{N}\left(t\right)=\zeta_{1}\left(t\right)=\zeta_{2}\left(t\right)=\cdots =\zeta_{N}\left(t\right)=\xi \left(t\right)\\ p=q \end{array}\right.$$

The choice of basis functions (such as Fourier series) is more flexible, and the basis function can be expressed as

(9a) $$\xi_{i}\left(t\right)=\left[1,\sin\left(\pi t\right),\cos\left(\pi t\right),\cdots ,\sin\left(p\pi t\right),\cos\left(p\pi t\right)\right]$$

When the power series is selected, the following equation exists:

(9b) $$\xi_{i}\left(t\right)=\left[1,t,t^{2},\cdots ,t^{p-1}\right]$$

p is related to the length of $\left[t_{b}∼t_{e}\right]$. When $\left[t_{b}∼t_{e}\right]$ is shorter, the number of expansion terms may be smaller, and the basis function type weakly affects the fitting function.

$X\left(t\right)$ and $F\left(t\right)$ can be converted into Equations (10) and (11) by means of Equations (6) and (7).

(10) $$X\left(t\right)=S\left(t\right)\alpha$$

(11) $$F\left(t\right)=S\left(t\right)\beta$$

where $S\left(t\right)$ is defined as a basis function matrix, which is expressed as

(12) $$S\left(t\right)={\left[\begin{array}{cccc} \xi \left(t\right) & 0 & \cdots & 0\\ 0 & \xi \left(t\right) & \cdots & 0\\ \vdots & \vdots & \ddots & \vdots \\ 0 & 0 & \cdots & \xi \left(t\right) \end{array}\right]}_{N\times \left(N\times p\right)}$$

α is the displacement response combination coefficient vector, and β is the external load combination coefficient vector, and they are expressed as

(13) $$\alpha ={\left\{\begin{array}{cccc} \alpha_{1} & \alpha_{2} & \cdots & \alpha_{N} \end{array}\right\}}^{T}$$

(14) $$\beta ={\left\{\begin{array}{cccc} \beta_{1} & \beta_{2} & \cdots & \beta_{N} \end{array}\right\}}^{T}$$

$\dot{X}\left(t\right)$ can be solved by means of Equation (10).

(15) $$\dot{X}\left(t\right)=\dot{S}\left(t\right)\alpha$$

where

(16) $$\dot{S}\left(t\right)={\left[\begin{array}{cccc} \dot{\xi }\left(t\right) & 0 & \cdots & 0\\ 0 & \dot{\xi }\left(t\right) & \cdots & 0\\ \vdots & \vdots & \ddots & \vdots \\ 0 & 0 & \cdots & \dot{\xi }\left(t\right) \end{array}\right]}_{N\times \left(N\times p\right)}$$

$X\left(t\right)$, $F\left(t\right)$, and $\dot{X}\left(t\right)$ can be expressed by means of Equations (10)–(15) as expressions of the known basis function matrices and their combination coefficients, which can be utilized to solve the corresponding responses or external loads.

### 2.3. Solving External Loads to Be Identified

The substitution of Equations (10) and (11) and Equation (15) into Equation (4) results in the following equations:

(17) Aα=Ββ

(18) $$A={\left.\left[D\left(t\right)M\dot{S}\left(t\right)-\dot{D}\left(t\right)MS\left(t\right)+D\left(t\right)CS\left(t\right)\right]\right|}_{t_{b}}^{t_{e}}+\int_{t_{b}}^{t_{e}}\left[\ddot{D}\left(t\right)M-\dot{D}\left(t\right)C+D\left(t\right)K\right]S\left(t\right)dt$$

(19) $$Β=\int_{t_{b}}^{t_{e}}D\left(t\right)S\left(t\right)dt$$

where A represents the coefficient matrix related to the structural responses, and Β represents the coefficient matrix related to the loads. The known $D\left(t\right)$ and $S\left(t\right)$ are selected, and A and ***B*** of Equation (17) can be calculated by means of Equations (18) and (19), respectively.

In an actual structure, some structural responses or external loads can be acquired by means of a reasonable arrangement and layout of sensors. Any structural response or external load can be decomposed into known and unknown parts. A,Β, α, and β of Equation (17) are expressed as

(20) $$\left[\begin{array}{cc} A_{k} & A_{n} \end{array}\right]\left\{\begin{array}{c} \alpha_{k}\\ \alpha_{n} \end{array}\right\}=\left[\begin{array}{cc} {Β}_{k} & {Β}_{n} \end{array}\right]\left\{\begin{array}{c} \beta_{k}\\ \beta_{n} \end{array}\right\}$$

where $A_{k}$ represents the known part of A, $\alpha_{k}$ represents the coefficient vector corresponding to $A_{k}$, $A_{n}$ represents the unknown part of A, $\alpha_{n}$ represents the coefficient matrix corresponding to $A_{n}$, ${Β}_{k}$ represents the known part of B, $\beta_{k}$ represents the coefficient vector corresponding to ${Β}_{k}$, ${Β}_{n}$ represents the unknown part of Β, and $\beta_{n}$ represents the coefficient vector corresponding to ${Β}_{n}$.

Equation (20) can be sorted by means of the rearrangement of $A_{k}\alpha_{k}$ and ${Β}_{n}\beta_{n}$ as

(21) $$\left[\begin{array}{cc} -{Β}_{n} & A_{n} \end{array}\right]\left\{\begin{array}{c} \beta_{n}\\ \alpha_{n} \end{array}\right\}=\left[\begin{array}{cc} {Β}_{k} & -A_{k} \end{array}\right]\left\{\begin{array}{c} \beta_{k}\\ \alpha_{k} \end{array}\right\}$$

Let

(22) $$\left\{\begin{array}{l} \Pi =\left[\begin{array}{cc} -{Β}_{n} & A_{n} \end{array}\right]\\ \theta =\left\{\begin{array}{c} \beta_{n}\\ \alpha_{n} \end{array}\right\}\\ \varphi =\left[\begin{array}{cc} {Β}_{k} & -A_{k} \end{array}\right]\left\{\begin{array}{c} \beta_{k}\\ \alpha_{k} \end{array}\right\} \end{array}\right.$$

Then, Equation (21) can be expressed as

(23) Πθ=φ

where Π represents the coefficient matrix composed of unknown external loads and displacement responses, θ represents the coefficient vector corresponding to Π, and φ represents the known vector composed of known external loads and displacement response. 

The number of collected displacement responses is no less than the number of loads to be identified, and the rational selection of $D\left(t\right)$ guarantees the full rank of columns of Π. The solution of Equation (23) can be expressed as

(24) $$\theta =\Pi^{+}\varphi$$

where $\Pi^{+}$ represents the Moore–Penrose generalized inverse of Π.

$\beta_{n}$, which is solved by means of Equation (24), is substituted into Equation (7) to obtain the load to be identified. The algorithm procedure for load identification is summarized in the Algorithm 1 below.
**Algorithm 1:** The solution algorithm for load identification.**Input**: mass matrix ***M***, damp matrix ***C***, stiffness matrix ***K***, p, the length of time sub-intervals Δ*t*, virtual function ***D***(*t*), basis function ***ξ***, number of time sub-intervals n_t_, known displacement ***x***_k_, known force ***f***_k_, *i =* 1.**Output**: ***f***_n.*i*_ , *t*_e,*i*_, *t*_b,*i*_.1: Solve Equation (9) for ***ξ***, on the basis of p;2: Solve Equation (12) for ***S***(*t*), on the basis of ***ξ***;3: **Repeat**;4: *t*_e,*i*_ = *t*_b,*i*_ + Δ*t*;5: Solve Equation (6) for ***α***_k,*i*_, on the basis of ***x***_k_, *t*_e,*i*_, *t*_b,*i*_, and ***ξ***;6: Solve Equation (7) for ***β***_k.*i*_, on the basis of ***f***_k_, *t*_e,*i*_, *t*_b,*i*_, and ***ξ***;7: Solve Equation (18) for ***A****_i_*, on the basis of *t*_e,*i*_, *t*_b,*i*_, ***M***, ***C***, ***K***, ***D***(*t*), and ***S***(*t*);8: Solve Equation (19) for ***B****_i_*, on the basis of *t*_e,*i*_, *t*_b,*i*_, ***D***(*t*), and ***S***(*t*);9: Solve Equation (22) for ***Π****_i_*, *ϕ_i_*, on the basis of ***α***_k,*i*_, ***β***_k.*i*_, ***A****_i_*, and ***B****_i_*;10: Solve Equation (24) for ***θ****_i_*, on the basis of ***Π****_i_*, and *ϕ_i_*;11: Solve Equation (7) for ***f***_n.*i*_ , on the basis of ***β***_k.*i*_, *t*_e,*i*_, *t*_b,*i*_, and ***ξ***;12: *i* = *i* + 1;13: End in the case of *i* = n_t_.

## 3. Numerical Simulation Cases

### 3.1. Structural Model

**Figure 2:**
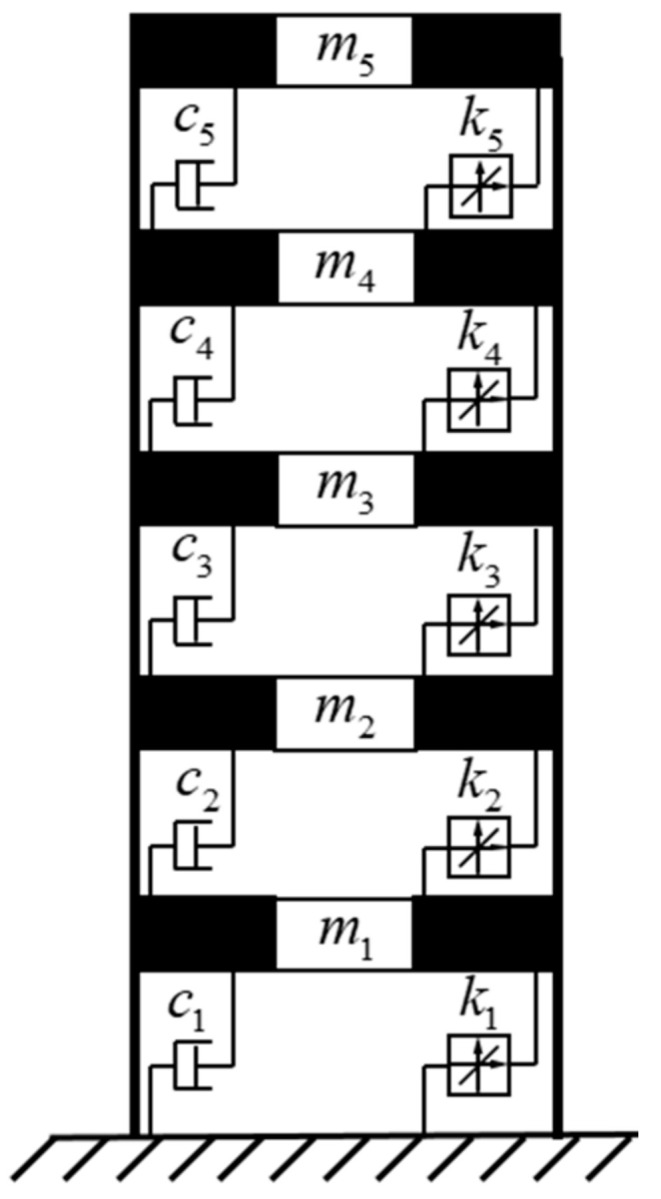
Numerical model.

To verify the effectiveness of our method, a five-layer shear numerical model (Figure 2) was established. As for each layer of structure, its mass, stiffness, and damping coefficient were 200 kg, 500,000 Nmm^−1^, and 150 Nsm^−1^, respectively. Its frequency parameters are shown in Table 1.

### 3.2. The Number of Basis Function Expansion Items and the Length of a Time Subinterval

Loads and structural displacement responses as signals to be identified or collected are fitted by means of the basis function in the time sub-interval $\left[t_{b}∼t_{e}\right]$. The longer $\left[t_{b}∼t_{e}\right]$ is, the more the signal amplitude and frequency change with time and the more items of the basis function are necessary for fitting these signals, and a matrix with more dimensions can be solved. To reduce the number of dimensions of the solution matrix, the length of $\left[t_{b}∼t_{e}\right]$ should not exceed the signal period. A basis function with fewer items is necessary for fitting on the premise that the accuracy of the signal fitting is satisfied. The parts for which the signal fitting effect is poor are located at the signal peaks, and the signals change more gently near those peaks. To ensure the signal fitting effect, the fitting effect of signals shall be guaranteed first in the vicinity of these peaks. The selection of $\left[t_{b}∼t_{e}\right]$ is related to the frequency of the fitted signal. If it contains several frequencies, the signal changes more violently with the growth of its frequency. The fitting effect of the highest frequency signal should be guaranteed. When the highest frequency of the fitting signals is assumed as *f_r_*, the period is *T* = 1/*f_r_*. The length of $\left[t_{b}∼t_{e}\right]$ does not exceed *T*. The signal fitting was performed here in time intervals whose lengths are $\frac{T}{2},\frac{T}{4}$ and $\frac{T}{8}$, namely,

(25) $$t_{e}-t_{b}=\frac{T}{N_{f}}$$

where $N_{f}=2,4$, and 8.

The basis function is necessarily derivated in Equation (15) so that the convenience of derivation shall be considered for the selection of the basis function type. Here, the power and trigonometric series are taken as the basis functions which are expressed as follows:

Case 1: $\xi \left(t\right)=\left[1,t,t^{2},\cdots ,t^{p-1}\right]$
Case 2: $\xi \left(t\right)=\left[1,\sin\left(\pi t\right),\cos\left(\pi t\right),\cdots ,\sin\left(p\pi t\right),\cos\left(p\pi t\right)\right]$

where p represents the order of the basis function.

Load or response signals are classified as periodic and random signals, and the latter may be generated by the method of harmonic superposition. Thus, harmonic and triangular wave signals were only taken into account here. From the perspective of waveform characteristics, both types of signals are periodic, but the latter is not differentiable in some positions. Most of the load frequencies are low in civil engineering. Thus, the highest and lowest frequencies of harmonic signals are 20 Hz and 1 Hz, respectively, and the highest and lowest frequencies of triangle wave signals are 5 Hz and 1 Hz, respectively. 

On the basis of the frequency (*f_r_*) of load or response signals, the length of $\left[t_{b}∼t_{e}\right]$ is determined. Power or trigonometric series are taken as the basis functions to study the changing rules of the number of its items (p) and errors between real values and fitted values of load or response signals. The relative errors are calculated by

(26) $$R_{e}=\frac{\sqrt{\sum {\left[g_{i}\left(t\right)-{\bar{g}}_{i}\left(t\right)\right]}^{2}}}{\sqrt{\sum g_{i}{\left(t\right)}^{2}}}\times 100\%$$

where $g_{i}\left(t\right)$ and ${\bar{g}}_{i}\left(t\right)$ represent the real and fitted values of the *i*^th^ load or response signal, respectively, and *R_e_* represents the relative error between $g_{i}\left(t\right)$ and ${\bar{g}}_{i}\left(t\right)$.

**Figure 3:**
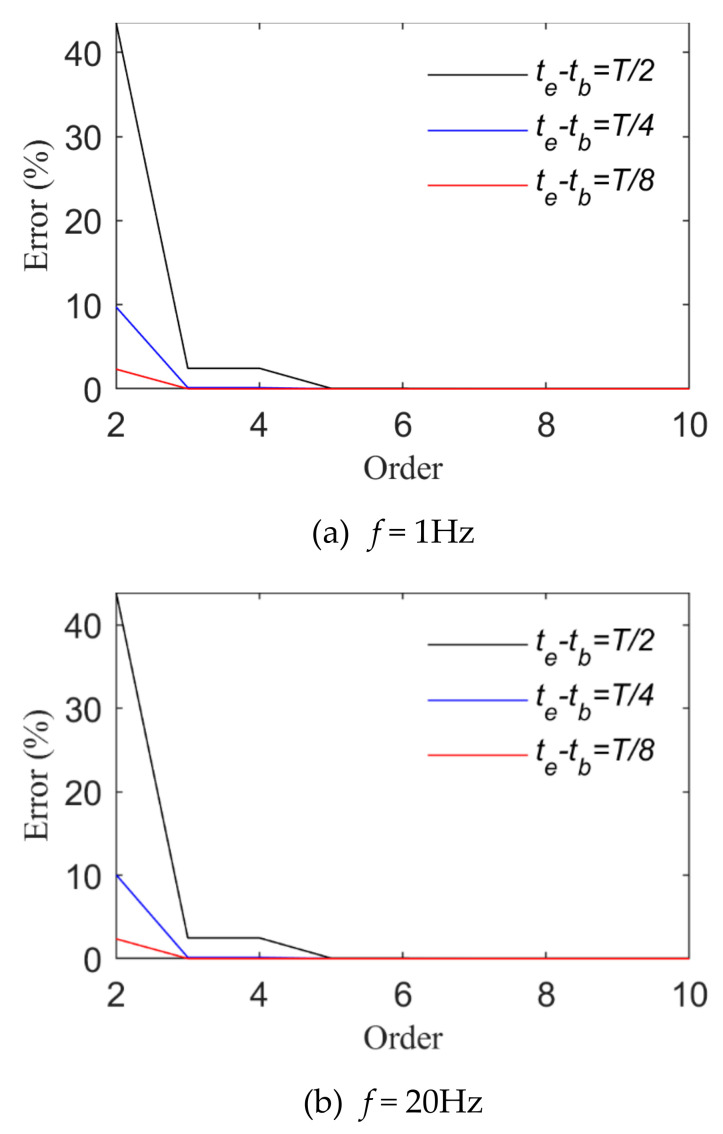
Fitting effects of harmonic loads (Case 1).

**Figure 4:**
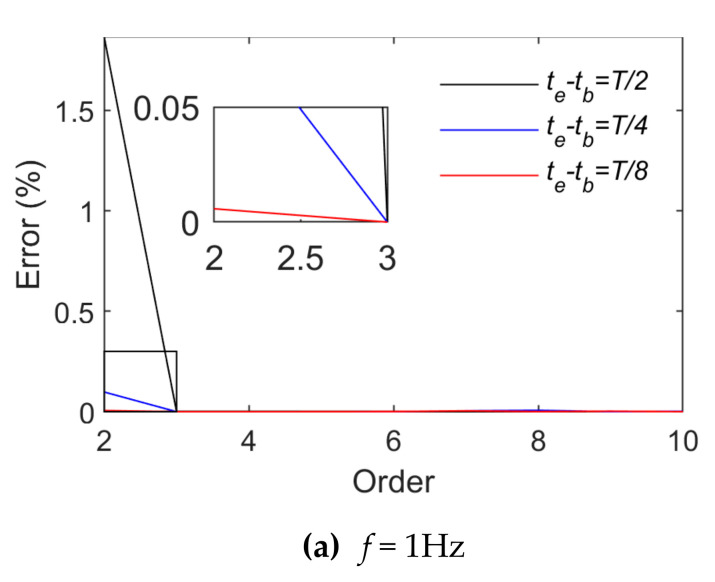

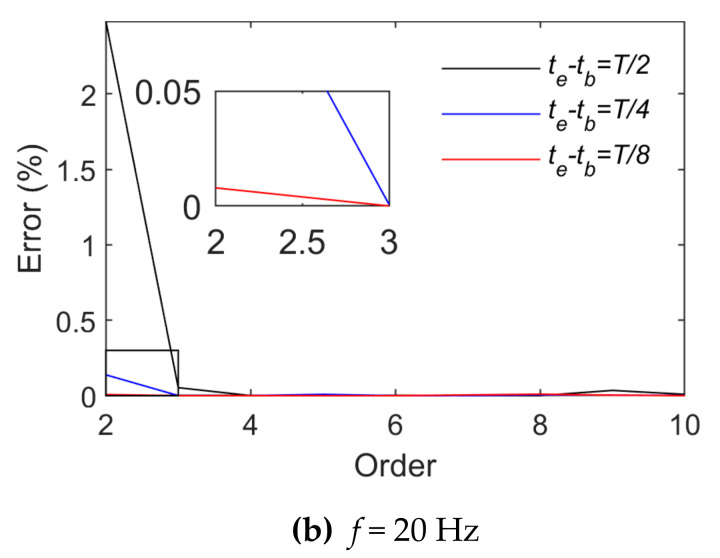
Fitting effects of harmonic loads (Case 2).

Figure 3 and Figure 4 show changes of the fitting order and *R_e_* in the case of fitting harmonic signals with power and trigonometric series, respectively. The longest time interval is a half-wavelength of the load cycle. The smaller the load frequency is, the straighter the load time history curve is, and the more the load time history curve changes. Thus, a higher order of the basis function is required when high-frequency signals are fitted. In view of harmonic signals, *f* is taken as 1 Hz or 20 Hz. When the length of their time intervals are $\frac{T}{2},\frac{T}{4}$ and $\frac{T}{8}$, the fitting relative errors basically converge to 0 when the power series or trigonometric series are expanded to the sixth order. Harmonic signals are fitted by means of power or trigonometric series. As harmonic signals are composed of trigonometric series, fewer orders are required for the application of trigonometric series (Figure 4 indicates that the relative error value of fitting basically converges to 0 after the fifth order of the trigonometric series).

**Figure 5:**
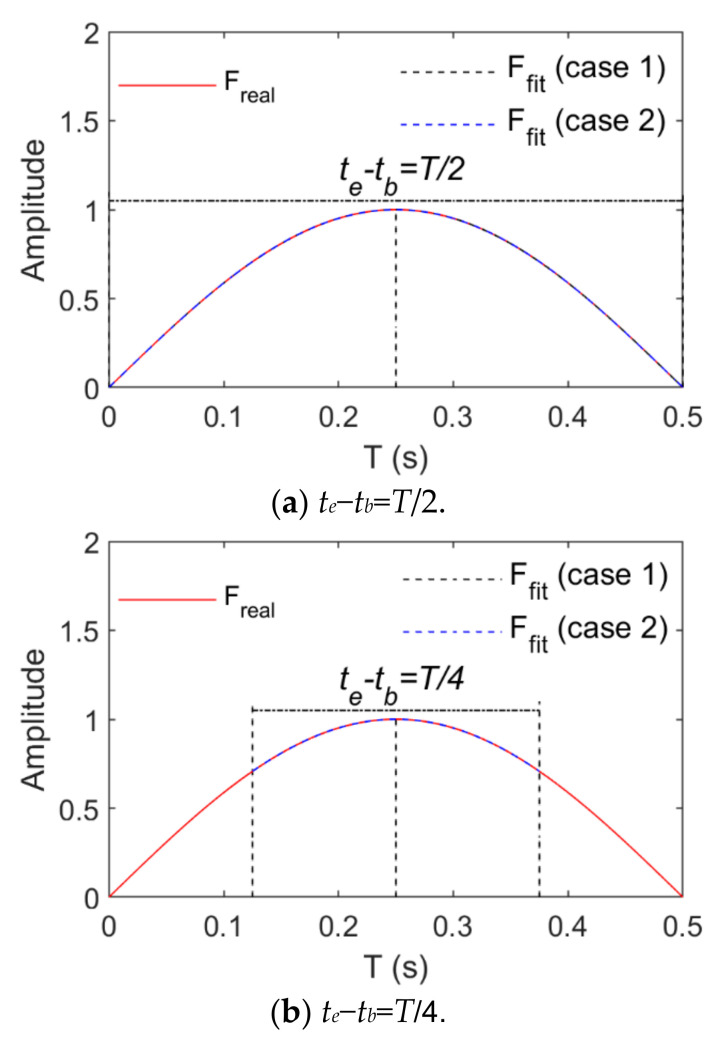

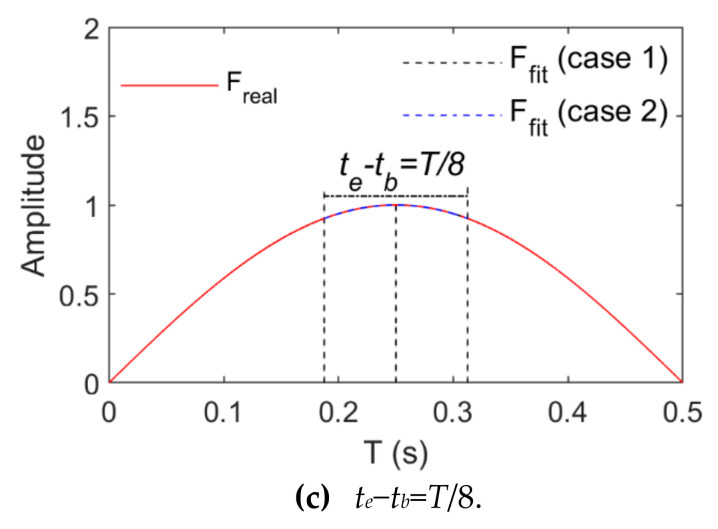
Comparison between real and fitting values of harmonic loads (*f* = 1 Hz).

**Figure 6:**
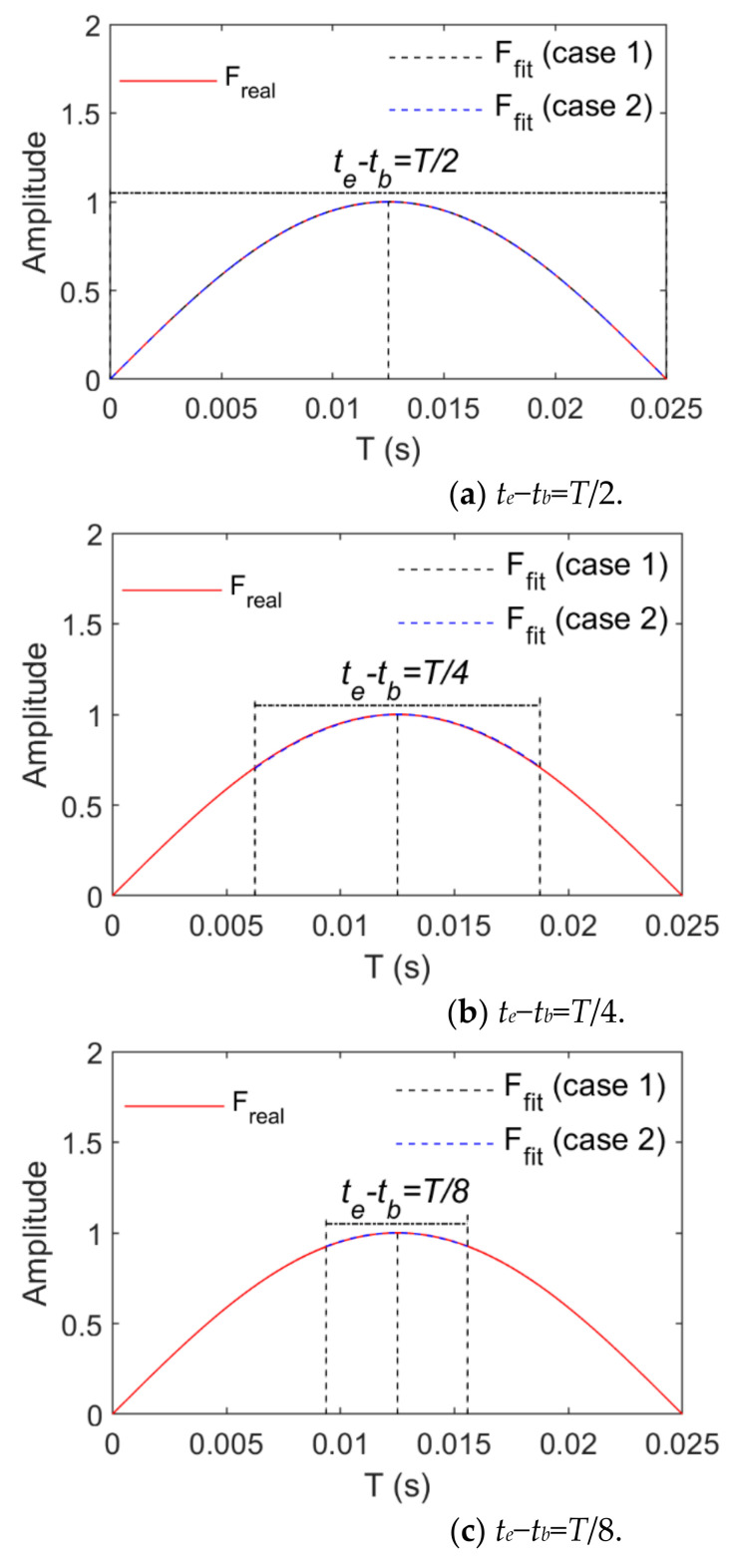
Comparison of real and fitting values of harmonic loads (*f* = 20 Hz).

Figure 5 and Figure 6 show a comparison between the real and fitting curves of harmonic signals (*f*: 1 Hz and 20 Hz and the tenth order) based on the application of power and trigonometric series, respectively. They indicate that the real and fitting curves are almost completely overlapped in $\left[t_{b}∼t_{e}\right]$.

**Figure 7:**
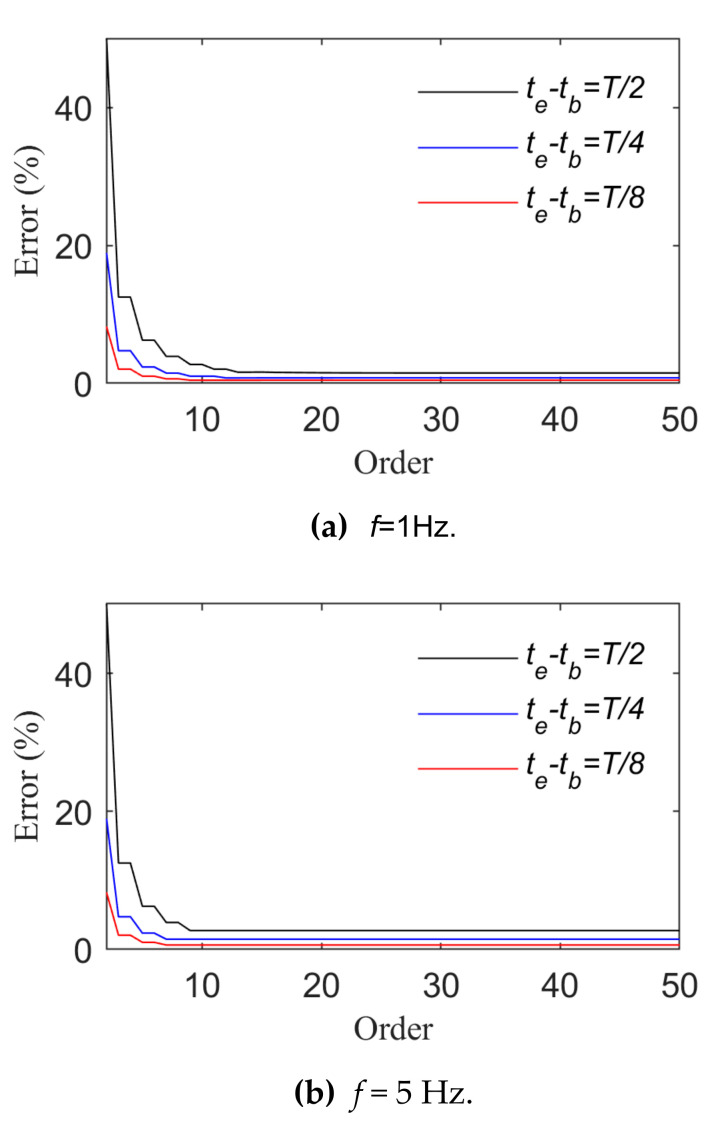
Fitting effects of triangular wave loads (Case 1).

**Figure 8:**
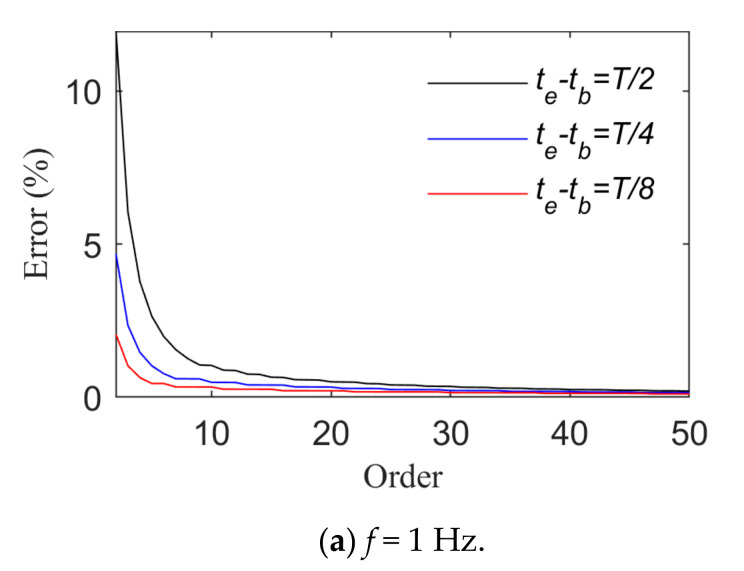

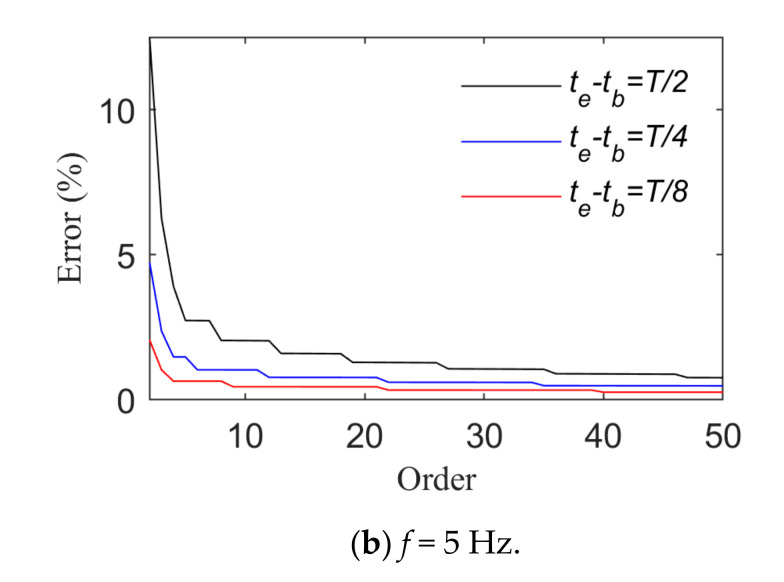
Fitting effects of triangular wave loads (Case 2).

Figure 7 and Figure 8 present the change rules of p and the relative errors between the real and fitting values when triangular wave signals are fitted by means of power and trigonometric series, respectively. They indicate that the fitting relative errors gradually decrease along with the growth of the order of the basis function, but they converge more slowly when the order of the basis function is above 10. When the order of the basis functions is the same, the fitting relative errors rise along with the growth of $\left[t_{b}∼t_{e}\right]$. A comparison of Figure 7 and Figure 8 indicates that triangular series rather than power series result in better effects of fitting triangular wave signals. As for triangular wave signals, fitting relative errors become basically stable when the length of $\left[t_{b}∼t_{e}\right]$ is taken as $\frac{T}{8}$ and the power series or trigonometric series expansion is performed to the tenth order.

**Figure 9:**
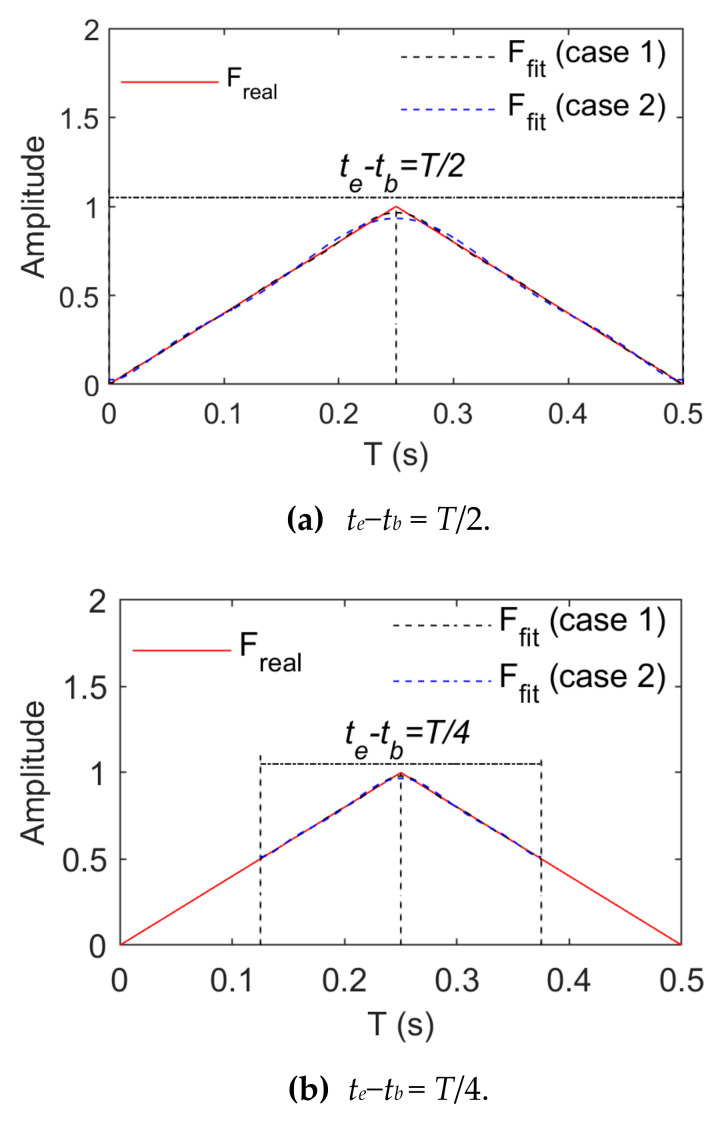

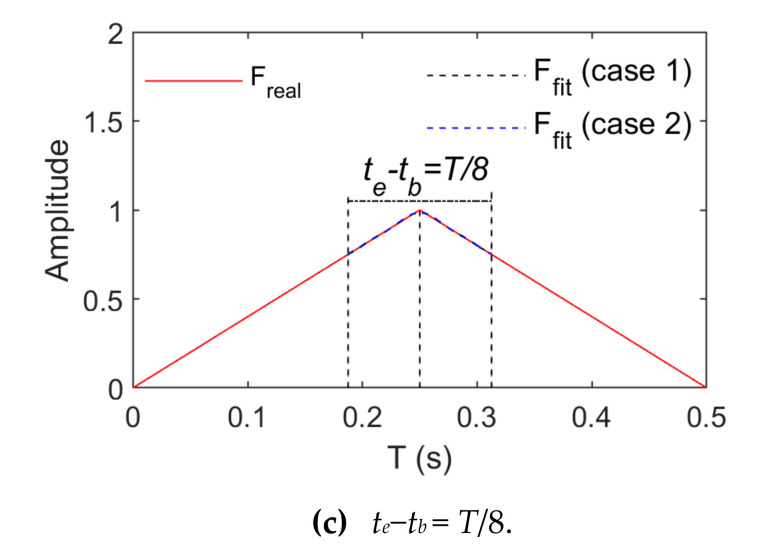
Comparison between real and fitting values of triangular wave loads (*f* = 1 Hz).

**Figure 10:**
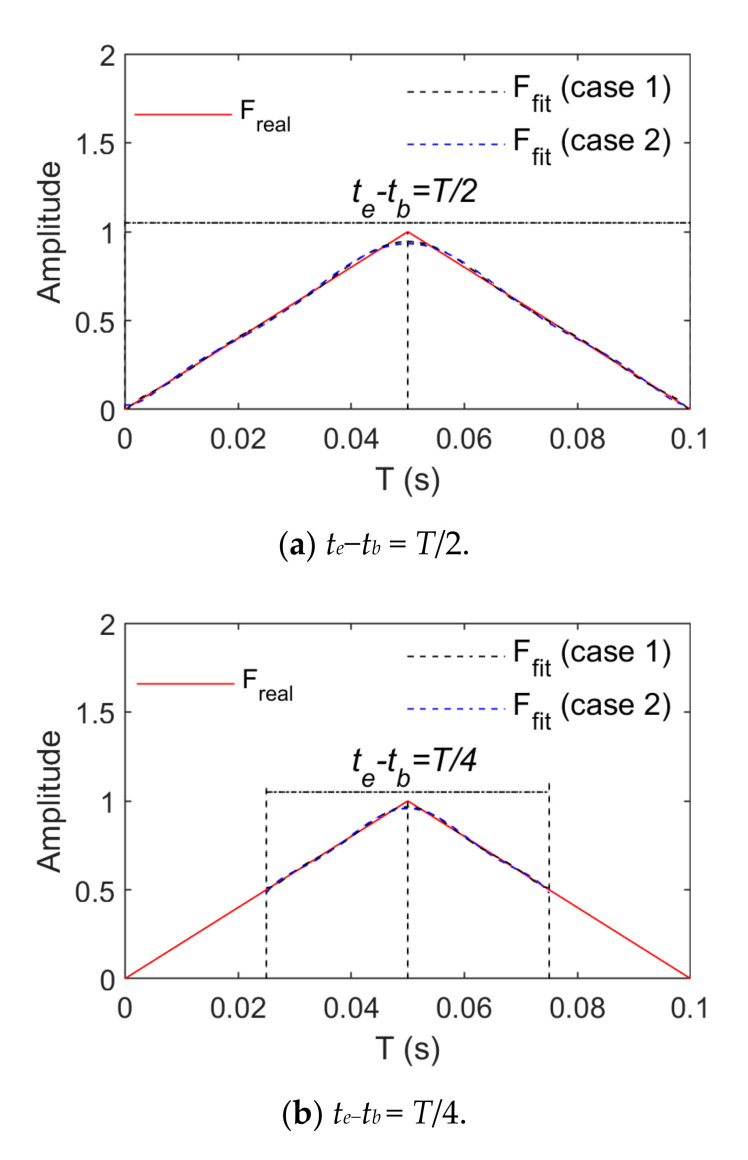

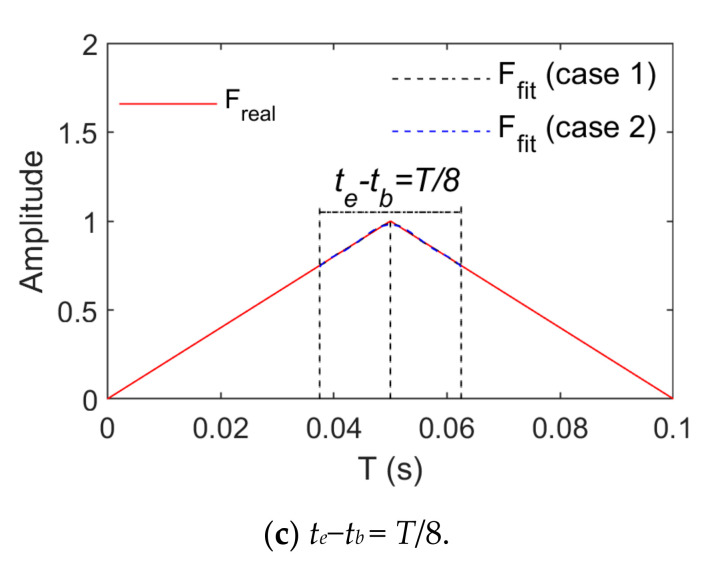
Comparison between real and fitting values of triangular wave loads (*f* = 5 Hz).

Figure 9 and Figure 10 show comparisons between the real and fitting curves of triangular wave signals (1 Hz and 5 Hz) in the case of the tenth order fitting by means of power and trigonometric series fitting, respectively. They indicate that real and fitting errors focus on peaks or troughs primarily due to non-differentiable properties at these positions of the triangular wave signals. Smooth basis function-based fitting only leads to approximation rather than accessibility. Growth of the number of fitting items of the basis function can reduce fitting errors but increase the amount of load identification calculations.

### 3.3. Load Identification Results

The loads acting on the structure take into account the random and the triangular wave load, respectively, and the external load acts at m5. On the basis of the requirements of our algorithm, the number of known structural responses is necessarily no less than the number of identification loads. Structural displacement responses were acquired here at Positions *m*_4_ and *m*_5_; additionally, the influence of different degrees of noise was taken into account in structural responses to consider the robustness of our algorithm to noise. Noise was added in accordance with Equation (27):

(27) $${\tilde{x}}_{i}\left(t\right)=x_{i}\left(t\right)+\eta \sqrt{\frac{{\left\|x_{i}\left(t\right)\right\|}_{2}^{2}}{l_{i}}}\psi \left(t\right)$$

where $x_{i}\left(t\right)$ and ${\tilde{x}}_{i}\left(t\right)$ represent the noise-free and noise displacement responses of the *i*^th^ degree of freedom, respectively; $l_{i}$ represents the length of $x_{i}\left(t\right)$ with discrete displacement responses; η represents the noise level, which was taken as 5% here; and $\psi \left(t\right)$ is a random sequence uniformly distributed between (-1~1), whose length is the same as that of $x_{i}\left(t\right)$.

The load duration was 10 s for our simulation. It was divided into several time sub-intervals ($\left[t_{b}∼t_{e}\right]$), whose lengths were $t_{e}=t_{b}+3\Delta t$ ($\Delta t=1/f_{s}$; $f_{s}$: the highest order frequency of load; adjacent sub-interval overlapping coefficient: τ=1). The basis functions are trigonometric and power series, which have 10 expansion terms. $D\left(t\right)$ is a degenerated virtual function ($d\left(t\right)$), which is expressed as

$$d\left(t\right)=\sin\left(\frac{\pi }{2}t\right)+\cos\left(\frac{\pi }{2}t\right)$$

For the characterization of the degree of similarity between identification and real loads, their correlation coefficient (ρ) is calculated according to Equation (28):

(28) $$\rho =\frac{\sum \left\{f_{i}\left(t\right)-E\left[f_{i}\left(t\right)\right]\right\}\left\{{\tilde{f}}_{i}\left(t\right)-E\left[{\tilde{f}}_{i}\left(t\right)\right]\right\}}{\sqrt{\sum {\left\{f_{i}\left(t\right)-E\left[f_{i}\left(t\right)\right]\right\}}^{2}\sum {\left\{{\tilde{f}}_{i}\left(t\right)-E\left[{\tilde{f}}_{i}\left(t\right)\right]\right\}}^{2}}}$$

where $f_{i}\left(t\right)$ and ${\tilde{f}}_{i}\left(t\right)$ represent the actual and identified external loads of the *i*^th^ degree of freedom, respectively; ρ represents the correlation coefficient of $f_{i}\left(t\right)$ and ${\tilde{f}}_{i}\left(t\right)$; and $E\left(\cdot \right)$ means averaging.

**Figure 11:**
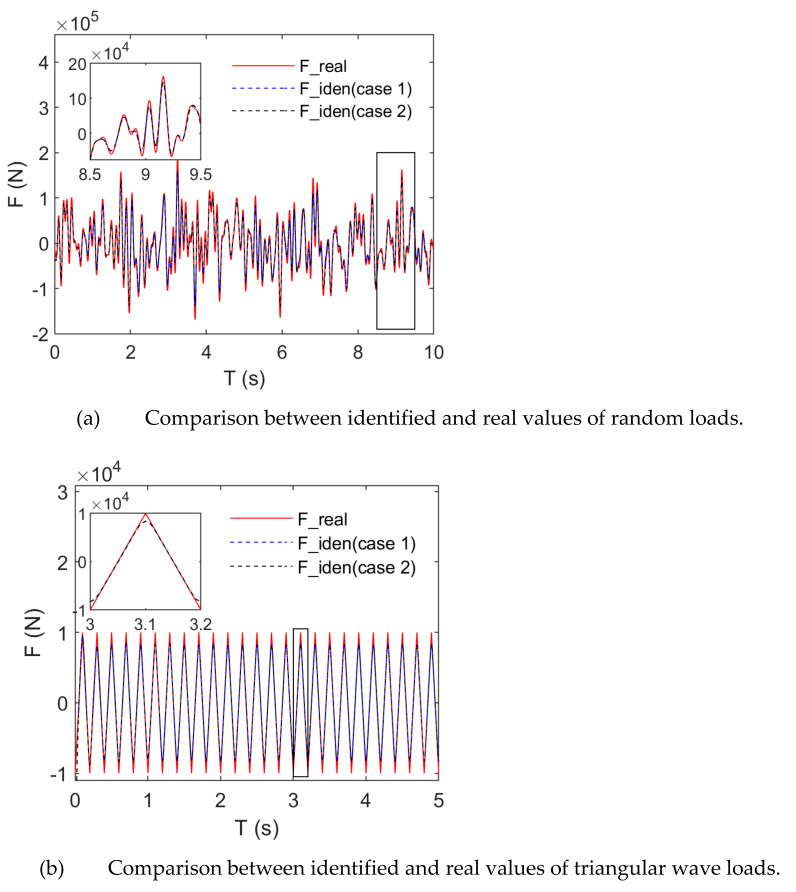
Comparison between identified and real values under noise-free conditions.

Figure 11 presents the identification effects of random and triangular wave loads under noise-free conditions. For a clear demonstration of the identification effects of triangular wave loads, Figure 11b only presents the history of the first 3 s of the triangular wave load, but the complete load time history data were utilized to calculate the correlation coefficient (Table 2). Figure 11 and Table 2 indicate that the shapes of random and triangular wave loads could be accurately identified when power series and trigonometric series were taken as basis functions. In the case of periodic triangular wave loads with a single frequency, the spectrum components were complex. The corresponding load identification results indicated that triangular wave loads, rather than random loads, could be better identified. Under noise-free conditions, the correlation coefficient between identified and real values was above 0.98 for random and triangular wave loads. From the perspective of load identification amplitudes, the identified amplitudes were slightly smaller than the real ones for random and triangular wave loads, and the triangular wave load errors focused on the non-differentiable positions. Identification load errors were mainly caused by amplitude errors under noise-free conditions. In the case in which the basis function was a power or trigonometric series, the random and triangular wave loads had basically the same identification effects. Our algorithm is not sensitive to the basis function type.

**Figure 12:**
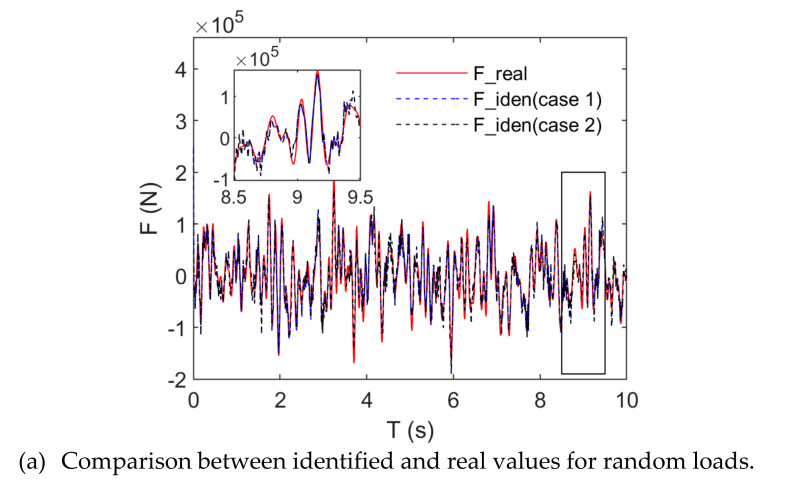

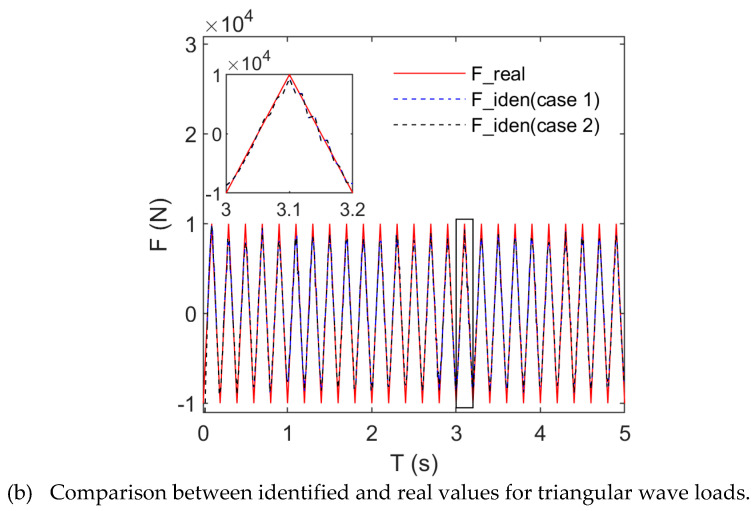
Comparison between identified and real values (noise level: 5%).

Figure 12 presents the identification effects and random and triangular wave loads when the noise level (5%) was taken into account. Figure 12a,b indicate that there was no remarkable irregularity for the identified and real amplitudes of random loads; conversely, the identified amplitudes were generally lower than the real amplitudes for triangular wave loads. Along with the identification errors for load amplitudes, the load frequencies also had identification errors. Figure 12 indicates that the identified values fluctuated slightly around their real values for random and triangular wave loads, namely, the spectra of the identification load also contained the high-frequency components of noise except for the real load frequency. Table 3 indicates that the correlation coefficient between the identified and real values was 0.92 for random loads. Figure 12a shows that the real random load waves can be identified accurately by means of our algorithm. The correlation coefficient was 0.99 for triangular wave loads, and the identified triangular wave loads were less affected by noise. A comparison of basis function-based identification effects of loads showed the similarity under noise-free conditions. In the case of the noise level (5%), our algorithm is not sensitive to the type of basis functions.

## 4. Experimental Verification

**Figure 13:**
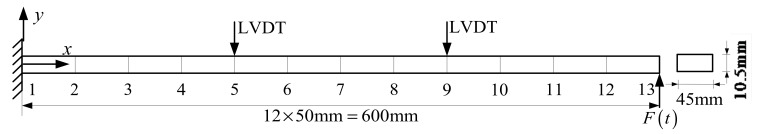
Schematic diagram of our cantilever beam tester.

**Figure 14:**
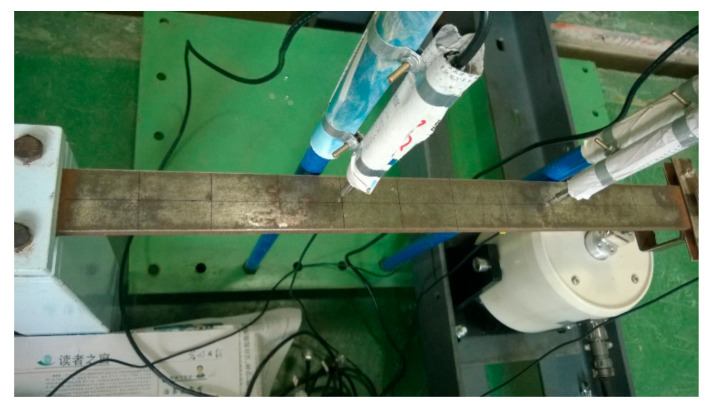
Experimental photo.

For further verification of the effectiveness of our algorithm, a steel cantilever beam excitation test was designed. The cantilever beam size was 600 mm × 45 mm × 10.5 mm. The vibration exciter was set at the cantilever end, and their interaction force was acquired by means of force sensors (signal acquisition frequency: 1000 Hz), which were located at the tripartite points of the cantilever beam. Figure 13 presents the layout of the vibration exciter and displacement sensors. Figure 14 shows a photo of our tester.

Random and periodic loads were taken into account in our test conditions. The random load spectra were within 20 Hz. A relatively ideal triangular wave could not be excited by means of our test exciter so that harmonic loads could act as periodic loads (frequency: 5 Hz). On the basis of the above numerical simulation, the basis function for the expansion of loads was the trigonometric series, and the number of expansion items was 10. $t_{e}-t_{b}=\frac{3}{f_{s}}$ (*f_s_* = 20 Hz) was taken here.

**Figure 15:**
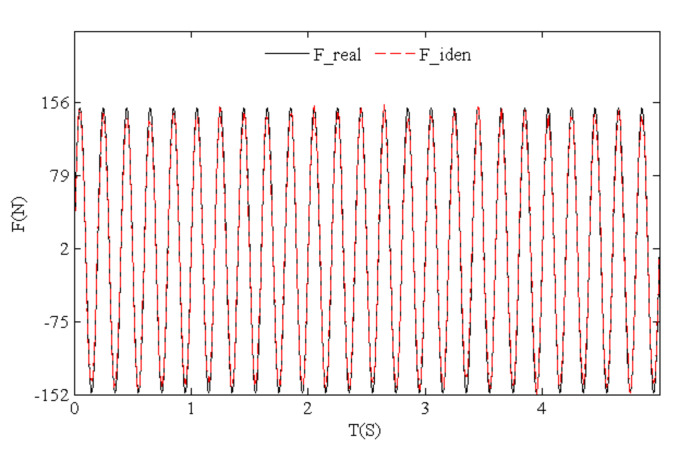
Time history of identified and real values for test harmonic loads.

**Figure 16:**
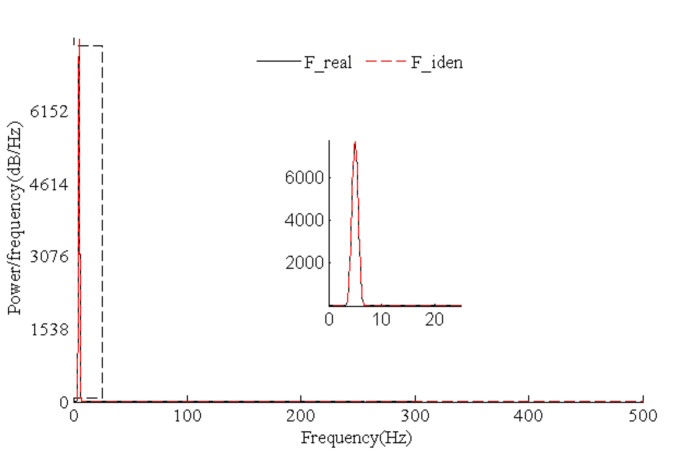
Identified and real PSDs for test harmonic loads.

Figure 15 presents the time history comparison of identified and real values for harmonic loads, which indicates that load identification errors focused on peaks or troughs. Such results are similar to simulated triangular wave load identification results. The identification effects were better for continuous and differentiable harmonic loads compared with triangular wave loads. The power spectral density diagram of load identification and real values (Figure 16) shows that the harmonic load frequency can be identified accurately by means of our algorithm.

**Figure 17:**
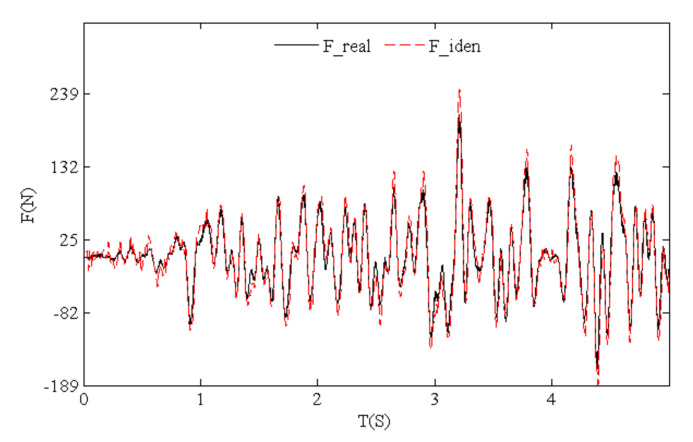
Time history of identified and real values for test random loads.

**Figure 18:**
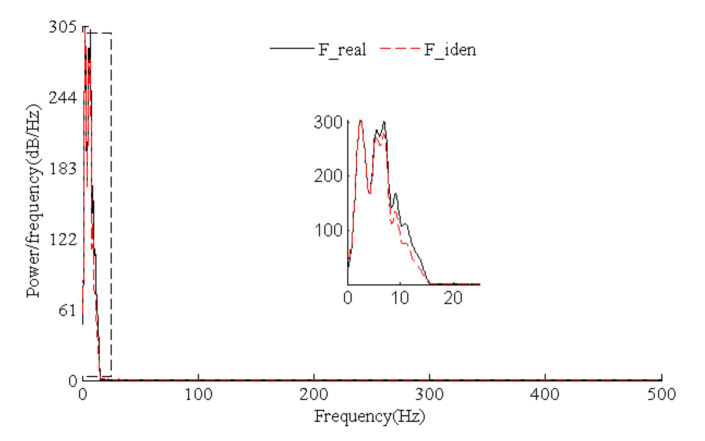
Identified and real PSDs for test random loads.

Figure 17 shows the time history comparison of identified and real values for random loads whose frequency components were complex. Identification effects of harmonic loads with a single frequency were better than those of the random loads. Figure 18 indicates that low-frequency components of random loads could be identified more easily. High-frequency noise caused a greater effect with the growth of the frequency, and the load identification errors gradually increased. However, the time history and power spectral density curves show that random loads were identified accurately by means of our algorithm.

Table 4 presents the load identification results, for which the relative error and the correlation coefficient were calculated by means of Equations (26) and (28), respectively. When the time subinterval is determined, the growth of the number (p) of expansion terms of the basis function can improve the load recognition effect when the calculation time is longer. Table 4 indicates that the relative error and the correlation coefficient of recognition results were 8.415% and 0.9306, respectively, when the length of the time subinterval was 0.15 s, and the number of expansion terms of the basis function was 10. Moreover, when the number of expansion terms of the basis function was 40, the relative error and the correlation coefficient rose by 16.0% ((8.415% – 7.065%)/8.415% × 100 = 16.0%) and just 0.3% ((0.9334 − 0.9306)/0.9306 × 100 = 0.3%). However, the calculation time increased by nearly 30 (5816/192 = 30.3) times. When the length of the time subinterval was 0.20 s and the number of expansion terms of the basis function was 20, the load identification results were similar to those in the case in which the length of the time subinterval was 0.15 s and the number of expansion terms of the basis function was 10. Namely, they had similar load recognition effects as the length of the time subinterval grew. It is necessary to increase the number of basis function expansion items simultaneously. The corresponding calculation time also increases.

If the length of the time sub-interval is determined and the number of expansion items of the basis function is p, the dimension of the solution matrix is N × p. The storage space of the computer increases, and higher computing power is required. If the length of the time sub-interval rises while the number of basis function expansion items remains unchanged, the amount of calculation in each time sub-interval remains the same, but the load fitting effect in the time interval becomes worse. Finally, a larger error in load identification will occur. As the length of time sub-intervals is increased, the number of time sub-intervals decreases, and the total calculation time also decreases. For example, when the number of open terms of the basis function was p = 10, and the time subinterval length was *t_e_* − *t_b_* = 0.15 s, the number of time subintervals was 67, and the total calculation time was 192 s. In the case of *t_e_*−*t_b_* = 0.2 s, the number of time sub-intervals was 50, and the total calculation time was 146 s. As for *t_e_* − *t_b_* = 0.2 s and *t_e_* − *t_b_* = 0.15 s, the ratio of their numbers of time subintervals was 0.75 (50/67 = 0.75), and the ratio of their total calculation durations was about 0.76 (142/192 = 0.76). These ratios are basically the same. In the case of *t_e_* − *t_b_* = 0.15 s and *t_e_* − *t_b_* = 0.2 s and p having any other value in Table 4, the average of the ratios of their total calculation durations was 0.78. This is also basically consistent with the ratio of the numbers of the time sub-intervals (0.75). Such a case indicates that the calculation time is primarily affected by the number of time subintervals when the number of expansion terms of the basis function remains constant.

The method described in [11] and our method were applied to identify loads by means of structural displacement responses. For our experiments, Table 5 presents the comparison between the load identification results of our method and the method in [11]. According to [11], the algorithm parameters were set as follows: time window length: 0.15 s (namely 150 time steps) and number of shape functions. Table 5 indicates that our method, in contrast to the method of [11], resulted in the relative error and the correlation coefficient of the recognition results of harmonic loads rising by 16.5% ((9.117% − 7.611%)/9.117% × 100 = 16.5%) and 4.6% ((0.9819 − 0.9385)/0.9385 × 100 = 4.6%), respectively. However, the calculation time of our method was reduced to about one quarter (178/720 ≈ 0.25) of that of [11]. As for the recognition results of random loads, our method, in comparison to that of [11], still caused the relative error and correlation coefficient to rise by 35.8% ((13.117% − 8.415%)/13.117% × 100 = 35.8%) and 15.3% ((0.9306 − 0.8070)/0.8070 × 100 = 15.3%), respectively; however, its calculation time was about one quarter (192/708 ≈ 0.27) of that of [11]. A comparison of the recognition results and calculation time of both methods indicates that our method can significantly improve recognition results of random loads, and our calculations were faster.

## 5. Conclusions

A load identification integration algorithm was proposed here based on structural displacement responses. Moreover, the effects of the time sub-interval length, the type of basis function for expansion of loads, and the number of expansion items of the basis function on identification were discussed in detail. Numerical simulations and experiments verified the effectiveness of our method. The conclusions are as follows:

(1) The length of the time sub-interval can be determined on the basis of *T*_min,_ which is defined as the period corresponding to the harmonic load with the maximum frequency. Loads to be identified are harmonic or first-order derivable random loads. When the length of the time sub-interval is no more than *T*_min_/2, and the loads are fitted by means of power or trigonometric series, the rational fitting order is 6. When loads to be identified are triangular wave loads whose time sub-interval length is no more than *T*_min_/8, and the loads are fitted by means of power or trigonometric series, the rational fitting order is 10. When the length of the time sub-interval is given, increasing the number of basis function expansion items can improve the load identification effects, but the calculation takes longer.
(2) When an appropriate time sub-interval length is taken, harmonic, triangular wave, and random loads can be effectively identified, and the calculation amount is small in the case of fitting by means of power or trigonometric series. The load identification effects are slightly better when using the trigonometric series-based fitted basis function.
(3) Noise can be suppressed to a certain extent by means of our load identification integral algorithm. Numerical simulations indicate that the correlation coefficient between identified and real loads can be above 0.9 in the case in which the measurement noise level is 5%. Our cantilever beam tests showed that the load frequency range can be identified accurately by means of our algorithm, and the load identification errors focus on peaks or troughs.

However, the following aspects were also necessarily improved in the case of the actual application of our method:

1. For a load with a wider frequency band, the identification effects of its high-frequency components will be improved.
2. Exploration of other load modeling methods should be carried out to further improve the accuracy of load identification.
3. A study on the effects of the type of a virtual function on load identification is required.

The solution to ill-conditioned matrices will be subsequently discussed, and the robustness of our algorithm to noise will be studied technically to further expand the identification of types of loads, such as impact and moving loads, on the basis of the application of our method. Additionally, we will attempt to apply our method in the areas mentioned in [40].

## Figures and Tables

**Table 1:** Model frequencies.

Order	1	2	3	4	5
Frequency (Hz)	2.27	6.61	10.42	13.39	15.27

**Table 2:** Noise-free load identification and real correlation coefficients.

Load Type	Basis Function Type	Correlation Coefficient
Random load	Power series	0.9881
	Trigonometric series	0.9985
Triangular wave load	Power series	0.9957
	Trigonometric series	0.9959

**Table 3:** Correlation coefficients of load identification and real values (noise level: 5%).

Load Type	Basis Function Type	Correlation Coefficient
Random load	Power series	0.9209
	Trigonometric series	0.9224
Triangular wave load	Power series	0.9952
	Trigonometric series	0.9924

**Table 4:** Load identification results of various methods.

*t_j_* − *t_i_* (s)	p	Relative Error (%)	Correlation Coefficient	Calculation Time (s)
0.15	4	62.768	0.0214	21
	6	13.238	0.9222	76
	10	8.415	0.9306	192
	20	7.783	0.9333	498
	40	7.065	0.9334	5816
0.20	4	69.841	0.4630	17.6
	6	17.432	0.8998	63.3
	10	9.478	0.9218	146
	20	8.271	0.9259	395
	40	8.209	0.9277	3849

**Table 5:** Load identification results of both methods.

Load Type	Method	Relative Error (%)	Correlation Coefficient	Calculation Time (s)
Harmonic load	Our method	7.611	0.9819	178
	Method of [11]	9.117	0.9385	720
Random load	Our method	8.415	0.9306	192
	Method of [11]	13.117	0.8070	708

## Data Availability

The data that support the finding of this study are available from the corresponding author upon reasonable request.
